# Evaluating three approaches to binary event-level agreement scoring. A reply to Friedman (2020)

**DOI:** 10.3758/s13428-020-01425-0

**Published:** 2020-07-23

**Authors:** Raimondas Zemblys, Diederick C. Niehorster, Kenneth Holmqvist

**Affiliations:** 1 Šiauliai University, Šiauliai, Lithuania; 2 Lund University Humanities Lab, Lund University, Lund, Sweden; 3 Department of Psychology, Lund University, Lund, Sweden; 4 Institute of Psychology, Nicolaus Copernicus University in Torun, Torun, Poland; 5 Department of Psychology, Regensburg University, Regensburg, Germany; 6 Department of Computer Science and Informatics, University of the Free State, Bloemfontein, South Africa

Recently, Friedman (2020) published a letter in which he claims there are three errors and two problems in our paper “gazeNet: End-to-end eye-movement event detection with deep neural networks” (Zemblys et al., 2019). Here we respond to these claims by Friedman, namely that improper data were used for Zemblys et al., (2019) and that performance was improperly evaluated.

Let us first recap what we presented in Zemblys et al., (2019). gazeNet is a method that takes an existing eye-movement data set that has been labeled (through hand-coding or by any other means) and trains a classifier to reproduce this event coding. The goal of gazeNet, as for any machine learning-based classifier, is to produce coding similar to what it observed during training. As such, the performance of classifiers like gazeNet is evaluated on other labeled data that was not seen during training, and the classifier is said to perform well if it is able to produce high agreement with the testing set (i.e., similar coding as the testing set). As such, the classifier can be trained on any input data, regardless of its quality, since the success of a classifier is determined by its performance on the testing set. In Zemblys et al., (2019), we used the procedure we proposed and trained a specific classifier using part of the *lund2013-image* data set (Larsson et al., 2013, see “Data” section in Zemblys et al., (2019) for detailed description), which was then evaluated on another unseen part of the *lund2013-image* data set, as well as the *GazeCom* (Starsev et al., 2016, 2017) and *humanFixationEvaluation* (Hooge et al. 2018, containing data from Hessels et al. 2016a).

## Data quality

The first error and the two problems that Friedman (2020) discusses are issues of data quality. Specifically, Friedman notes that:

1. Several of the files from the *lund2013-image* data set used in Zemblys et al., (2019) provided gaze data sampled at 200 Hz instead of the 500 Hz assumed by us and reported by the authors of the data set. Friedman(2020, “Error 1”)
2. The intersample intervals in the *lund2013-image* data set were not constant. Friedman (2020, “Problem 1”)
3. The trajectories of saccades in the *lund2013-image* data set were not smooth but contained discontinuities. Friedman (2020, “Problem 2”)

Furthermore, in footnote 4, Friedman (2020) speaks of problems with the other data sets, *GazeCom* and *humanFixationEvaluation*, used by Zemblys et al., (2019) to evaluate the performance of gazeNet. Specifically, Friedman (2020) claims that there are misclassification errors in these data sets.

Our response to the claims by Friedman (2020) regarding the above problems and errors in Zemblys et al., (2019) is the following: First, it is correct that some of the files in the *lund2013-image* data set provide gaze data sampled at 200 Hz instead of 500 Hz. Furthermore, as observed by Friedman (2020), the intersample intervals in this data set were indeed not constant (as is frequently observed in data from SMI eye trackers, see e.g., Hessels et al., 2015; Niehorster et al., 2020c) and discontinuities were present in the trajectories of saccades (see also, Holmqvist and Blignaut, 2020), which, to our experience, they usually are in data from the SMI HiSpeed system with which this data set was recorded. Given the above characteristics of the *lund2013-image* data set, Friedman (2020) claims that we used improper data in Zemblys et al., (2019) and that our use of this data gives a “basis for concern” (p. 2). While it remains unclear what the concern would be, his mentioning of errors and problems in the gazeNet paper insinuates that he is concerned that the conclusions of our study are invalid. We disagree with this concern, and with the notion that the data we used was improper for our study, or that it was an error or problem to use this data. Friedman (2020) has neither made an effort to back up his claim by showing what the problem would be, nor investigated whether it has a relevant impact on the analyses and conclusions we reported. Furthermore, Friedman’s logic that imperfect data imply that a study’s results are erroneous is invalid. For instance, while saccades recorded with two different eye trackers (e.g., an SMI and an EyeLink) may appear dissimilar because their waveforms have different characteristics, the similarities between saccades of the two eye trackers are likely much larger than the similarities between either of the saccades and a fixation or PSO. This would mean that the datasets we used, despite containing imperfections in Friedman’s (2020) view, would still enable the training of a robust and generalizable event classifier and a valid evaluation of its performance and the performance of other algorithms. The analyses reported below reinforce our argument. Despite disagreeing with Friedman’s logic that some characteristics of the eye-tracker data we used are problematic, we nonetheless, for the sake of argument, will throughout this paper refer to these characteristics of our training and evaluation data sets as “imperfections”.

Second, since the goal of the gazeNet classifier was to reproduce the coding observed during training, below we argue that the presence of imperfections such as non-constant intersample intervals and discontinuities in saccade trajectories do not invalidate the results reported in Zemblys et al., (2019), but instead provide an important test case for the robustness of our approach. In the “Replication using only 500-Hz data” section below, we furthermore report on a newly trained gazeNet classifier using only 500-Hz data, and on new performance evaluations of all algorithms on a testing set consisting of only 500-Hz data. These new results furthermore underscore that the inclusion of 200-Hz data during training and evaluation had a minimal impact on the results reported in Zemblys et al., (2019). Their exclusion did not change the paper’s conclusions.

Third, the imperfections in the data sets used for training and evaluation should logically only lead to reduced performance of the evaluated event classifiers, which would be reflected in lower agreement between the event classifiers’ output and the (hand-coded) event labels in the evaluation data sets. This is logically expected because a deep-learning classifier such as gazeNet that is trained on a data set with specific characteristics (such as the imperfections in the *lund2013-image* data set) might perform less well on other data sets that do not contain these same characteristics. Yet the agreement scores reported in Zemblys et al., (2019) were very high despite the imperfections in the data and dissimilarities of the data set on which Zemblys et al., (2019) was trained and some of the data sets used for evaluation. This shows that the deep learning-based event classification method presented in Zemblys et al., (2019) is robust to various imperfections in the training and evaluation data sets and, importantly, that the results reported in Zemblys et al., (2019) are not invalidated by the presence of these imperfections in the training data set. Friedman’s (2020) reasoning that the presence of imperfections in the training data means that this data was “improper” for how it was used in Zemblys et al., (2019) is thus invalid, as is his claim that use of this data constitutes errors and problems in the gazeNet paper.

Fourth, robustness to imperfections in the input data is an attribute of an event classifier that is of significant importance. Temporal and spatial noise, as well as systematic imperfections such as the saccade discontinuities in data from the SMI HiSpeed 1250, are present in eye-tracking data from most systems in most applications (see, e.g., Hessels et al., 2015, 2018; Niehorster et al., 2020a, b; Holmqvist and Blignaut 2020) and therefore must be dealt with efficiently and robustly. As we emphasize in the gazeNet paper, we believe that a major limitation of traditional hand-crafted algorithms is that they only work for certain data sets or only when certain conditions (e.g., a certain maximum level of RMS-S2S imprecision) are met. As we have shown (Zemblys et al., 2018, 2019), machine learning-based approaches to creating event classifiers may be able to surmount this limitation of traditional methods.

Fifth, as we explicitly state in Zemblys et al. (2019, p. 859), gazeNet is not meant to be a specific event classifier that one can download and use out of the box. Instead, the goal of the gazeNet paper was to develop a procedure for training end-to-end classifiers for eye-tracking data. The job of this procedure is to train a classifier that produces similar classification of input data as would have been produced by the process that the classifier observed during training. Simply put, if human coders were able to code a segment even if it contained serious imperfections (cf., Hooge et al., 2018), a good event classifier should be able to reproduce this coding. The agreement scores reported in Zemblys et al., (2019) show that a specific classifier trained using the gazeNet procedure and tested on challenging data sets succeeded well in this job. Note that it is important to use suitably classified input data for training the classifier before using it in a practical application because the goal of such classifiers is to obtain similar classification (high agreement), not to attain some unknowable “correct” classification. We therefore reiterate here our advice that users should train their own classifier that is optimally suited for their own purpose and data sets. This flexibility afforded by our approach is a strength that, as we have shown, enables automatically constructing event classifiers that function well also for data with various imperfections.

## Unfair evaluation

A further “error” discussed by Friedman et al. (2020, “Error 2”) is that it is unfair to compare an event classifier trained on a data set with other classifiers that were not trained on or developed using that data set. In our case specifically, Friedman claims that gazeNet had an unfair advantage when it was evaluated on the *lund2013-image-test* data set. Our evaluation however used established best practice of both the machine learning and the eye-movement classification fields (e.g., Larsson et al.,, 2013; Startsev et al.,, 2019a; Friedman et al.,, 2018). Specifically, first, evaluation was performed on a subset of the complete *lund2013-image* data set (called the testing set) that the gazeNet algorithm had not seen during the training process. Second, we have evaluated gazeNet and the other algorithms also on other data sets (*GazeCom* and *humanFixationEvaluation*) that neither gazeNet nor the other algorithms had seen before, and reported good performance of the gazeNet classifier also on these data sets. Together, these two standard procedures ensure that good performance reported on the testing data set is not the mere result of overfitting (e.g., the classifier learned the peculiarities of the particular training data set while the competitor algorithms did not have that chance). Instead, using this procedure, good performance across data sets indicates that the classifier is robust to the peculiarities of individual data sets and shows that it is able to generalize to other substantially different data sets.

## Event-level agreement analysis

There is one final “error” discussed by Friedman et al. (2020, “Error 3”), which concerns the implementation of our per-event event-related agreement analysis and the logic behind it. Friedman (2020) claims that this error inflates the per-event event-level agreement scores reported in Tables 7 and 8 of Zemblys et al., (2019). In this section, we investigate this claim.

First, the claim that the code posted online by us to compute per-event event-level agreement scores does not match the intended procedure described in the method section of Zemblys et al., (2019) may be due to an oversight in our method description. Specifically, in Tables 7 and 8 in Zemblys et al., (2019), we report event-level (binary) agreement scores for fixation, saccade, and PSO events separately, along with an overall agreement score in Table 7. Code implementing both per-event and overall agreement scores was made available at https://github.com/r-zemblys/ETeval. The procedure for the overall agreement score was described in the “Novel event-level evaluation” section on page 845 of Zemblys et al., (2019), but we have discovered that the procedure for per-event (binary) event-level agreement score computation was not described in the methods section. We therefore provide here the description of this procedure, as an addendum to this section of Zemblys et al. (2019, p. 845):

Besides evaluating overall event-level agreement for all events (fixations, saccades and PSOs) together, it is also informative to examine the extent of agreement for each event individually. To do so, the following procedure was used. We first turn the ground truth and algorithm event streams into binary streams denoting events of interest and other events. Below, we will refer to these as positive events (the event under evaluation, e.g., a fixation) and negative events (the other events that are not under evaluation, e.g., saccades and PSOs), respectively. Adjacent events of the same type are merged. We then perform the same matching procedure as above, i.e., matching events in the ground truth stream with those in the algorithm stream that have the most overlap. The remaining unmatched positive events are then labeled as false negatives or false positives, depending on whether they occur in the ground truth or algorithm event streams. Unmatched negative events are labeled as true negatives, so that these events do not penalize the per-event-agreement score for misclassification of events other than the event that is being evaluated. This relabeling procedure also enables all input events to count towards the agreement score, while enabling the outcome of the evaluation procedure to be summarized by a Cohen’s kappa score.

### Analysis of binary event matching

Friedman (2020) claims (“Error 3”) that when evaluating the per-event event-level agreement score between two streams of events, it is incorrect to count unmatched negative events as true negatives as that increases the Cohen’s kappa score, indicating higher agreement. Instead, Friedman (2020) claims that these unmatched negative events should be counted as false negatives or false positives, depending on whether they occur in the ground truth or algorithm event streams so as to drive Cohen’s kappa lower. Friedman (2020) furthermore states that we could have used an F1 score to assess per-event event-level agreement and that this, due to the nature of how an F1 score is calculated, would have avoided the problem of how to deal with unmatched negative events.

We agree with Friedman (2020) that the procedure we followed in Zemblys et al., (2019) to count unmatched negative events as true negatives may inflate the Cohen’s kappa score. We however disagree with Friedman’s framing of this issue as a dichotomy between correct and incorrect, since different agreement evaluation approaches make different trade-offs to optimize for different aspects of evaluating event-level agreement, and thus merely provide a different view of agreement between two event streams. In this context, it is worth noting that despite that the development of event-level agreement scores has started only very recently in the eye-tracking field, there are already multiple different approaches available (see, e.g., Hooge et al.,, 2018; Zemblys et al.,, 2019; Hoppe and Bülling 2016; Kothari et al.,, 2020; Startsev et al.,, 2019a, b). Each of these approaches found in the literature only provides a different view of agreement between two event streams and may be appropriate to use in some situations but not in others.

We furthermore underline here that the occurrence of unmatched negative events in per-event event-level agreement evaluation necessitates a change in the procedure to compute the Cohen’s kappa score. These unmatched negative events must be relabeled to include them in the per-event event-level Cohen’s kappa score since unmatched events do not feature in the confusion matrix underlying the measure. In Zemblys et al., (2019), we opted for the approach of relabeling unmatched negative events as true negatives in order to be able to include all events from the two streams in the evaluation of agreement. This decision enabled us to keep the agreement evaluation procedure for the per-event case as similar as possible to the procedure used for the overall agreement score, thereby providing a per-event Cohen’s Kappa score that remained comparable to the overall agreement Cohen’s kappa. We opted to not use the F1 score for assessing per-event agreement for the same reason of being able to report a per-event event-level agreement score that is comparable to the overall event-level agreement score.

Another approach we could have chosen for dealing with unmatched negative events was proposed by Friedman (2020), i.e., to penalize the agreement score by relabeling unmatched negative events as false negatives and false positives. However, we do not think that his proposal to penalize the event-level agreement score for mismatches in negative events, i.e., events other than the one for which the algorithm is being evaluated, provides a desirable view of algorithm performance for the event-type under evaluation.

Besides the approach we originally employed in Zemblys et al., (2019) of counting unmatched negative events as true negatives and the approach suggested by Friedman (2020) of counting unmatched negative events as false negatives and false positives (we will refer to this approach as the “unmatched as error” approach), there is a possible third approach when relaxing the restriction that all input events must count in the resulting agreement score. Specifically, our proposed procedure is to disregard the unmatched negative events in the calculation of the agreement score, so that these events which are not of interest neither increase nor penalize agreement between two event streams (see also, Startsev et al., 2019b). Here, we will refer to this as the “disregarding unmatched” approach. We think the disregarding unmatched approach, for our purposes, best reflects agreement on only the positive events, which is what we aimed to assess with the per-event agreement score. The potential drawback of this approach is that not all input events count in the evaluation of agreement and that the procedure therefore yields an approximate Cohen’s kappa score. For reference, for the per-event agreement scores reported in Table 7 of Zemblys et al. (2019, p. 855), unmatched negative events made up between 1.0% and 1.7% of all negative events for fixations and saccades, and between 7.0% and 11.7% for PSOs. These unmatched negative events would be ignored using the disregarding unmatched approach.

To provide the reader with insight into the impact of these three different approaches to per-event event-level agreement scores, we have augmented the ETEval code available at https://github.com/r-zemblys/ETeval to also produce agreement scores using the disregarding unmatched and unmatched as error approaches. We used this updated version to recompute the per-event event level agreement scores that were presented in Tables 7 and 8 in Zemblys et al., (2019). The per-event agreement scores in Table 7 of Zemblys et al. (2019, p. 855) can be compared to Table 1 in this paper and the values in their Table 8 (p. 856) to Table 2 here. For the latter table, like in Zemblys et al., (2019), three different data sets, *lund2013-image-test*, *GazeCom* and *humanFixationEvaluation* were used to evaluate the performance of gazeNet along with three other algorithms: Nyström and Holmqvist (2010, referred to as NH2010), Friedman et al. (2018, referred to as MNH), and two versions of Zemblys et al. (2018, referred to as IRF and IRF-spec).

**Table 1:** Per-event event-level Cohen’s kappa values for each event class when treating unmatched negative events as true negatives (cf. Table 7 of Zemblys et al. 2019, p. 855), when disregarding them, and when counting unmatched negative events as errors (false positives or false negatives)

Comparison	Unmatched as true negative			Disregarding unmatched			Unmatched as error		
	Fixations	Saccades	PSO	Fixations	Saccades	PSO	Fixations	Saccades	PSO
Experts									
testSet	0.966	0.983	0.844	0.965	0.983	0.836	0.952	0.956	0.715
gazeNet vs:									
trainSet	0.972	0.978	0.654	0.971	0.977	0.623	0.949	0.944	0.446
valSet	0.978	0.978	0.790	0.978	0.978	0.772	0.956	0.956	0.609
testSet RA	0.966	0.966	0.774	0.966	0.966	0.760	0.936	0.946	0.631
testSet MN	0.973	0.970	0.795	0.973	0.969	0.783	0.943	0.943	0.661

**Fig. 1:**
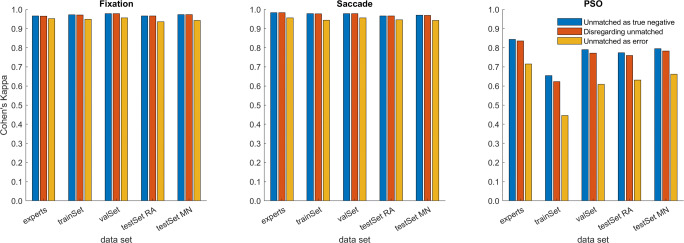
Visualization of the data in Table 1. Per-event event-level Cohen’s kappa values for each event class for the three different agreement evaluation approaches

Data are plotted in Fig. 1

**Table 2:** Per-event event-level Cohen’s kappa values for each event class when treating unmatched negative events as true negatives (cf. Table 8 of Zemblys et al. 2019, p. 856), when disregarding them, and when counting unmatched negative events as errors (false positives or false negatives)

Data set	Algorithm	Unmatched as true negative			Disregarding unmatched			Unmatched as error		
		Fixations	Saccades	PSO	Fixations	Saccades	PSO	Fixations	Saccades	PSO
lund2013-image-test	gazeNet	**0.959**	**0.947**	**0.776**	**0.957**	**0.945**	**0.762**	**0.840**	**0.889**	**0.632**
	IRF	0.780	0.848	0.616	0.755	0.842	0.582	0.558	0.743	0.405
	IRF-spec	0.783	0.844	0.693	0.759	0.836	0.668	0.563	0.733	0.503
	MNH	0.837	0.759	0.598	0.814	0.741	0.561	0.577	0.579	0.375
	NH2010	0.639	0.798	0.350	0.563	0.789	0.289	0.231	0.697	0.099
GazeCom	gazeNet	0.915	**0.845**	-	0.908	**0.835**	-	**0.753**	0.714	-
	IRF	0.844	0.779	-	0.831	0.774	-	0.665	**0.727**	-
	IRF-spec	0.843	0.774	-	0.829	0.768	-	0.664	0.718	-
	MNH	**0.921**	0.771	-	**0.914**	0.765	-	0.747	0.709	-
	NH2010	0.647	0.745	-	0.577	0.735	-	0.259	0.660	-
HumanFixation-	gazeNet	0.700	-	-	0.650	-	-	0.340	-	-
Classification	IRF	**0.707**	-	-	**0.664**	-	-	**0.376**	-	-
	IRF-spec	0.701	-	-	0.657	-	-	0.368	-	-
	MNH	0.389	-	-	0.248	-	-	–0.149	-	-
	NH2010	0.477	-	-	0.355	-	-	–0.103	-	-
genSet	gazeNet	**0.918**	**0.884**	**0.719**	**0.907**	**0.877**	**0.700**	**0.678**	**0.760**	**0.522**
	IRF	0.719	0.702	0.436	0.663	0.688	0.390	0.347	0.572	0.200
	IRF-spec	0.720	0.701	0.465	0.663	0.687	0.419	0.350	0.571	0.220
	MNH	0.792	0.606	0.340	0.742	0.579	0.287	0.381	0.401	0.106
	NH2010	0.326	0.543	0.087	0.149	0.509	0.014	–0.405	0.325	–0.162

**Fig. 2:**
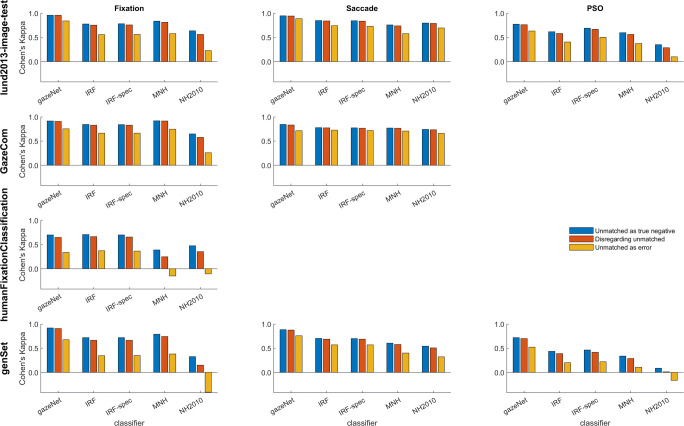
Visualization of the data in Table 2. Per-event event-level Cohen’s kappa values for each event class for the three different agreement evaluation approaches. Kappa values are shown for five event classification algorithms in each panel and, across the rows of panels, for four data sets

Kappa values are shown for five event classification algorithms and four data sets. The highest-scoring algorithm in each cell is printed in bold, while the runner-up is underlined. Data are plotted in Fig. 2

Comparing the scores in the “Unmatched as true negative” sections of Tables 1 and 2 to the values reported in Zemblys et al., (2019) reveals that they are identical, confirming that the changes made when augmenting the ETEval code did not alter the output of the algorithm. It can furthermore be seen that the strategy of disregarding unmatched events, which we argue is the approach that is most suitable for our aim of evaluating agreement on the event class under evaluation, produced event-level scores that are very similar in magnitude to those originally published in Zemblys et al., (2019), and identical in terms of relative ranking of algorithm performance. The unmatched as error approach suggested by Friedman (2020) on the other hand leads to systematically much lower agreement scores, that in a few cases even become negative. We think that this reflects significant overpenalization on the part of this agreement evaluation scheme.

## Replication using only 500-Hz data

In this section, we assess the impact of the inclusion of trials from the *lund2013-image* data set that were recorded at 200 Hz on the results reported in Zemblys et al., (2019). We do so in two ways. First, we retrained the gazeNet classifier using only data from trails recorded at 500 Hz. Second, we evaluated the performance of the retrained gazeNet classifier, as well as all the algorithms used by Zemblys et al., (2019), on a testing data set containing only data recorded at 500 Hz. In this section, data sets containing only 500 Hz data, or classifiers trained on only 500 Hz data will be identified by the suffix *-500*.

To retrain gazeNet, the same training and validation sets were used as in Zemblys et al.’s (2019, see their Table 9), except that trails recorded at 200 Hz were removed. Specifically, data from the files TL48_img_Europe_labelled_RA.mat and TL48_img_ Rome_labelled_RA.mat were removed from the training set, and the files UH47_img_Europe_labelled_RA.mat and UH47_img_Europe_labelled_MN.mat from the validation set. This left 36.6s of data in the training set, compared to 43.8s originally, and 2 × 19.8*s*, compared to 2 × 23.8*s* in the validation set (see Table 1 in, Zemblys et al. 2019). The same procedure as reported in Zemblys et al., (2019) was used to train this new classifier. Specifically, first a new gazeGenNet was trained using only the 500-Hz data. The resulting gazeGenNet-500 was then used to generate a new genSet-500 and gazeNet-500 was then trained using this genSet-500. All model and training parameters for gazeGenNet-500 and gazeNet-500 were the same as in Zemblys et al., (2019), except that 1500 instead of 2000 training steps were used when training gazeGenNet-500 because the removal of the trials recorded at 200 Hz left less input data.

Tables 3 (cf. Table 7 of Zemblys et al. 2019) and Table 4 (cf. Table 8 of Zemblys et al. 2019) report comparisons between the event-level agreement scores achieved with the original gazeNet classifier and with gazeNet-500. As can be seen in Tables 3 and 4, gazeNet-500 performed nearly identical to gazeNet across all overall and per-event event level agreement scores. The single exception is that gazeNet-500 performed substantially better than gazeNet on PSOs in the training set (Table 3). These results show that the inclusion of 200-Hz data when training gazeNet had only minimal impact on the results reported in Zemblys et al., (2019), and underscores that training on a data set that contained imperfections is not an error.

**Table 3:** Per-event event-level Cohen’s kappa for each event class, overall event-level Cohen’s kappa and event error rate (EER) for gazeNet (trained including trials recorded at 200 Hz, cf. Table 1) and gazeNet-500 (trained excluding these trials)

Data set	gazeNet					gazeNet-500				
	Fixations	Saccades	PSO	All	EER^′^	Fixations	Saccades	PSO	All	EER^′^
trainSet/-500	0.971	0.977	0.623	0.840	10.20	0.982	0.973	0.733	0.869	8.05
valSet/-500	0.978	0.978	0.772	0.881	7.10	1.000	1.000	0.774	0.909	6.02
testSet RA/-500	0.966	0.966	0.760	0.861	7.60	0.966	0.955	0.749	0.859	8.04
testSet MN/-500	0.973	0.969	0.783	0.871	6.85	0.958	0.944	0.754	0.854	7.08

**Fig. 3:**
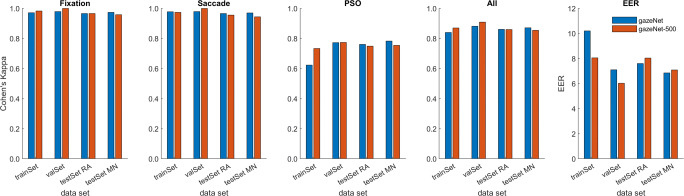
Visualization of the data in Table 3. Per-event event-level Cohen’s kappa values for each event class and event error rate (EER) values for gazeNet (trained including trials recorded at 200 Hz) and gazeNet-500 (trained excluding these trials). Unmatched negative events were disregarded

EER^′^ = EER*100. Unmatched negative events were disregarded for the per-event values. For gazeNet, each of the data sets on which performance was tested were as originally used in Zemblys et al., (2019), while for gazeNet-500, data sets excluding 200 Hz data were used. Data are plotted in Fig. 3

**Table 4:** Per-event event-level Cohen’s kappa values for each event class for gazeNet (trained including trials recorded at 200 Hz, cf. Table 2) and gazeNet-500 (trained excluding these trials). Kappa values are shown for four data sets

Data set	gazeNet			gazeNet-500		
	Fixations	Saccades	PSO	Fixations	Saccades	PSO
lund2013-image-test/-500	0.957	0.945	0.762	0.954	0.936	0.745
GazeCom	0.908	0.835	-	0.909	0.829	-
humanFixationClassification	0.650	-	-	0.719	-	-
genSet/-500	0.907	0.877	0.700	0.939	0.898	0.647

**Fig. 4:**
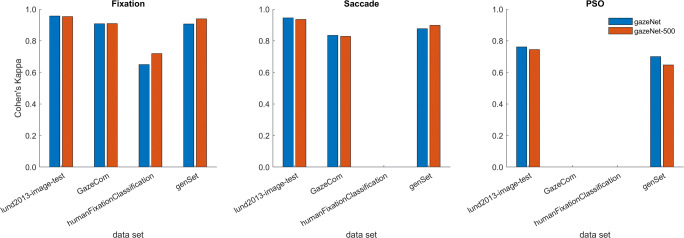
Visualization of the data in Table 4. Per-event event-level Cohen’s kappa values for each event class for gazeNet (trained including trials recorded at 200 Hz) and gazeNet-500 (trained excluding these trials). Kappa values are shown for four data sets. Unmatched negative events were disregarded

Unmatched negative events were disregarded. For gazeNet, the original data sets from Zemblys et al., (2019) (including 200-Hz data) were used, while for gazeNet-500, data sets lund2013-image-test-500 and genSet-500 excluding 200-Hz data were used. Data are plotted in Fig. 4

We furthermore evaluated gazeNet-500, as well as the original gazeNet classifier and the other three algorithms on only the trials in the *lund2013-image-test* data set that were recorded at 500 Hz. Specifically, the files UL47_img_konijntjes_labelled_RA.mat and UL47_img_konijntjes_labelled_MN.mat were removed from the testing data set. Table 5 presents a comparison of the per-event event level agreement scores obtained with all classifiers on the *lund2013-image-test* data set including trials recorded at 200 Hz as originally reported in Table 8 of Zemblys et al., (2019), to the *lund2013-image-test-500* set excluding these 200 Hz trials. It is readily appreciated from the table that while some of the classifiers (MNH and NH2010 especially) produced higher agreement scores for *lund2013-image-test-500* than *lund2013-image-test*, the ranking of classifiers by agreement was not affected. It is furthermore seen that the agreement scores for the machine learning-based classifiers was almost identical for the two data sets, showing that they were robust to the inclusion of 200-Hz data.

**Table 5:** Per-event event-level Cohen’s kappa values for each event class for testing sets including trials recorded at 200 Hz (*lund2013-image-test*, cf. Table 2) and excluding these trials (*lund2013-image-test-500*). Kappa values are shown for six classifiers

Algorithm	lund2013-image-test			lund2013-image-test-500		
	Fixations	Saccades	PSO	Fixations	Saccades	PSO
gazeNet-500	0.952	0.939	0.748	0.954	0.936	0.745
gazeNet	0.957	0.945	0.762	0.956	0.940	0.769
IRF	0.755	0.842	0.582	0.788	0.861	0.625
IRF-spec	0.759	0.836	0.668	0.788	0.855	0.700
MNH	0.814	0.741	0.561	0.858	0.803	0.635
NH2010	0.563	0.789	0.289	0.591	0.821	0.318

**Fig. 5:**
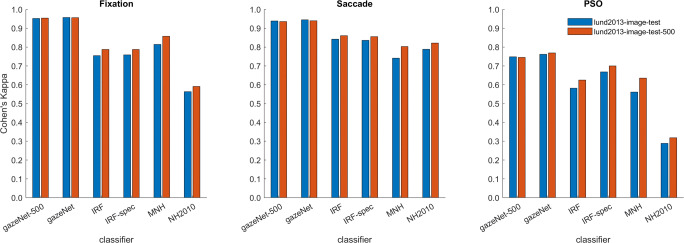
Visualization of the data in Table 5. Per-event event-level Cohen’s kappa values for each event class for testing sets including trials recorded at 200 Hz (*lund2013-image-test*) and excluding these trials (*lund2013-image-test-500*). Kappa values are shown for six classifiers. Unmatched negative events were disregarded

Unmatched negative events were disregarded. Data are plotted in Fig. 5

## Conclusions

In summary, we have discussed the claims of Friedman (2020) of errors and problems with our gazeNet paper (Zemblys et al., 2019). First, we have discussed that the imperfections in the training and testing data sets discussed by Friedman (2020) underscore that our procedure to create event classifiers through deep learning methods is sound. Indeed, our results consistently show that the gazeNet architecture delivers an event classifier that is robust to these imperfections and flexible enough to generalize and attain high agreement when evaluated on different data sets. Second, we have pointed out that the evaluations in the gazeNet paper were carried out according to standard practice on separate unseen data from the same set as the training set, and on completely different data sets provided by other research groups. Third, we have discussed and evaluated three different approaches to binary event-level agreement scoring. We found that our suggested new approach of disregarding missed classifications of events other than the event being evaluated yielded an agreement score that best reflects agreement on only the event of interest, which was our purpose for this procedure. This approach also yields only minimal differences from the agreement scores using the approach of Zemblys et al., (2019), and does not lead to different conclusions than those reported in that paper. We find that, in contrast, the approach suggested by Friedman (2020) significantly overpenalizes the agreement score in this situation. Last, we report results that are nearly identical to those in Zemblys et al., (2019) both when training gazeNet with only 500-Hz data and when evaluating the performance of all classifier algorithms with only 500-Hz data. These findings underscore that the “errors” in the data sets indicated by Friedman (2020) had minimal impact on our results and did not affect our conclusions.

## Appendix

The data in Tables 1–5 are presented in figure form in Figs. 1–5.
